# Threats and knowledge gaps for ecosystem services provided by kelp forests: a northeast Atlantic perspective

**DOI:** 10.1002/ece3.774

**Published:** 2013-09-15

**Authors:** Dan A Smale, Michael T Burrows, Pippa Moore, Nessa O'Connor, Stephen J Hawkins

**Affiliations:** 1 The Laboratory, Marine Biological Association of the United KingdomCitadel Hill, Plymouth, PL1 2PB, UK; 2 Ocean and Earth Science, National Oceanography Centre, University of Southampton, Waterfront CampusEuropean Way, Southampton, SO14 3ZH, UK; 3 Department of Ecology, Scottish Association for Marine Science, Scottish Marine InstituteOban, Argyll, PA37 1QA, UK; 4 Institute of Biological, Environmental and Rural Sciences, Aberystwyth UniversityAberystwyth, SY23 3DA, UK; 5 Centre for Marine Ecosystems Research, Edith Cowan UniversityJoondalup, 6027, WA, Australia; 6 School of Biological Sciences, Queen's University BelfastBelfast, BT9 7BL, UK; 7 School of Biological Sciences, University of SouthamptonSouthampton, SO17 1BJ, UK

**Keywords:** kelp forests, Laminariales, marine biodiversity, subtidal benthic habitats, temperate reefs.

## Abstract

Kelp forests along temperate and polar coastlines represent some of most diverse and productive habitats on the Earth. Here, we synthesize information from >60 years of research on the structure and functioning of kelp forest habitats in European waters, with particular emphasis on the coasts of UK and Ireland, which represents an important biogeographic transition zone that is subjected to multiple threats and stressors. We collated existing data on kelp distribution and abundance and reanalyzed these data to describe the structure of kelp forests along a spatial gradient spanning more than 10° of latitude. We then examined ecological goods and services provided by kelp forests, including elevated secondary production, nutrient cycling, energy capture and flow, coastal defense, direct applications, and biodiversity repositories, before discussing current and future threats posed to kelp forests and identifying key knowledge gaps. Recent evidence unequivocally demonstrates that the structure of kelp forests in the NE Atlantic is changing in response to climate- and non-climate-related stressors, which will have major implications for the structure and functioning of coastal ecosystems. However, kelp-dominated habitats along much of the NE Atlantic coastline have been chronically understudied over recent decades in comparison with other regions such as Australasia and North America. The paucity of field-based research currently impedes our ability to conserve and manage these important ecosystems. Targeted observational and experimental research conducted over large spatial and temporal scales is urgently needed to address these knowledge gaps.

## Introduction

Rapid environmental change is a threat to the functioning of marine ecosystems. Increased temperature, storminess, and changes in the frequency and magnitude of extreme climatic events will influence the distribution of species, community structure, and ecosystem functioning (Harley et al. 2006; Brierley and Kingsford 2009). These changes are likely to degrade the ecological services that natural systems provide (Hoegh-Guldberg and Bruno 2010; Sunday et al. 2012). The upper layers of the global ocean have warmed at a rate of 0.1°C per decade since the mid-20th Century, albeit with pronounced regional and seasonal variability (Solomon et al. 2007). The NE Atlantic region represents a hot spot of warming, as temperatures have risen at rates of ∼0.3–0.8°C per decade (Hughes et al. 2010; Lima and Wethey 2012). Seawater temperatures off the west coast of the UK and Ireland are predicted to warm by a further ∼2°C by 2090 (relative to 1990, see Philippart et al. 2011), with major implications for marine ecosystems. Other human-derived stressors interact with regional-scale climate change in unpredictable and nonlinear ways to impact marine ecosystem structure and functioning (Wernberg et al. 2011). In developed regions, such as the NE Atlantic, fishing and exploitation of other living marine resources, including seaweeds, plus coastal land use have impacted nearshore ecosystems for centuries. Over the last 150 years, diffuse (e.g., eutrophication) and point source chronic pollution has increased, although recent control measures and de-industrialization in the last few decades have led to improvements. Therefore, the current ecosystem “baseline” is far from pristine and is to some degree a product of humankind's role as the dominant ecosystem engineer and keystone predator (sensu sliding baselines, Dayton et al. 1998). Intensifying anthropogenic impacts over recent decades, which will continue into future (Halpern et al. 2008), dictate that comprehensive understanding of ecosystem functioning and resilience is of growing importance. This knowledge is needed to enhance sustainability in the use of ecological goods and services that coastal zones provide.

Kelps (large seaweeds of the order Laminariales) dominate rocky reefs throughout the world's temperate seas (Steneck et al. 2002), where they provide ecosystem services to humans worth billions of pounds (Beaumont et al. 2008). Kelp forests support high primary productivity, magnified secondary productivity, and a three-dimensional habitat structure for a diverse array of marine organisms, many of which are commercially important. Dominant kelp genera vary across the world's temperate bioregions, from *Laminaria* in the North Atlantic to *Ecklonia* in the Indian Ocean through to *Macrocystis* in the Pacific and South Atlantic (Raffaelli and Hawkins 1996; Steneck et al. 2002). Despite differences in the dominant species, kelp forests over the world share some commonality in their structure and functioning. For example, dominant canopy-forming kelps influence their environment and other organisms, thereby functioning as “ecosystem engineers” (*sensu* Jones et al. 1994). By altering light levels (Wernberg et al. 2005), water flow (Rosman et al. 2007), physical disturbance (Connell 2003), and sedimentation rates (Eckman et al. 1989), kelps modify the local environment for other organisms. Moreover, through direct provision of food and structural habitat, kelp forests support higher levels of biodiversity and biomass than simple, unstructured habitats (Dayton 1985; Steneck et al. 2002), and in general, kelp forests are hugely important as fuels for marine food webs through the capture and export of carbon (Dayton 1985; Krumhansl and Scheibling 2012).

Kelp forests can be highly dynamic systems that exhibit pronounced spatiotemporal variability. Kelps are susceptible to physical, chemical, and biological changes in the marine environment so that significant reduction in kelp habitat over tens to hundreds of kilometers can occur within a year (Dayton et al. 1992; Edwards 2004; Wernberg et al. 2013). Kelp forests within systems influenced by upwellings or variable oceanic boundary currents may be particularly dynamic, compared with those in more stable systems. Key factors include light, which is in turn influenced by latitude, water clarity, epiphytes, and weather, as well as temperature, nutrient levels, the frequency and intensity of storms, and outbreaks of herbivores. Crucially, recovery from perturbations can progress once environmental conditions become favorable; most kelp species reach maturity within 1–6 years (Parke 1948; Kain 1975b), and entire kelp-associated communities can recover within 7–10 years (Christie et al. 1998). Indeed, the recovery of kelp canopies and their associated assemblages following physical disturbance can be very rapid, occurring within 3 years (Hawkins and Harkin 1985). However, the resilience of kelp forests to perturbation is being eroded through multiple, concurrent chronic and acute stressors. In many regions, herbivory (usually by sea urchins) has increased as a result of trophic cascade effects associated with the removal of large predators (Estes and Duggins 1995; Steneck 1998). Increased herbivore pressure can cause phase shifts from structurally and biologically diverse kelp forests to simple, depauperate barrens (Breen and Mann 1976; Hagen 1983; Norderhaug and Christie 2009). In Tasmania, the impacts of a climate-mediated range expansion of a sea urchin have been compounded by overfishing of large lobsters, which would otherwise have kept the urchin population boom in check and limited grazing pressure (Ling et al. 2009). Other kelp systems have been degraded following increased nutrient and sediment input from ever-expanding coastal cities (Connell et al. 2008) or following establishment of nonindigenous species (Irigoyen et al. 2011; Krumhansl et al. 2011). Moreover, changing climatic variables, including storm frequency (Byrnes et al. 2011), the magnitude of extreme thermal events (Wernberg et al. 2013), and increased seawater temperature (Serisawa et al. 2004) have recently been attributed to ecologically significant alterations in kelp forest structure and functioning.

This review is not intended to duplicate existing syntheses on the biology and ecology of kelp species (Kain 1979; Dayton 1985), the resilience of kelp forests to perturbation (Steneck et al. 2002), kelps as drivers of detrital food webs (Krumhansl and Scheibling 2012), or the likely responses of kelp and other macroalgae to global environmental change (Harley et al. 2012). The aims of the review are threefold: (1) to synthesize existing knowledge on the structure and functioning of kelp forests, and the ecosystem services they provide, in the NE Atlantic with specific focus on the UK and Ireland; (2) to identify current threats to kelp forests and to assess the likely responses of kelp species and their associated biodiversity to key environmental change stressors; and (3) to highlight pressing knowledge gaps and research priorities that will lead to improved understanding of the current and future role of kelp-dominated habitats within the wider ecosystem. This information will ultimately support decision-making processes and feed into adaptive management approaches, which are needed to ensure the sustainability and continued productivity of natural ecosystems faced with rapid environmental change.

## A Brief History

Quantitative research on UK kelp forests began over 60 years ago, following a demand from the Ministry of Supply to produce camouflage textiles and other goods from kelp-derived alginates during and after the Second World War (Parke 1948; Woodward 1951). In the early 1950s, attempts were made to quantify the total standing stock of kelp as a potential exploitable resource. The total biomass of subtidal kelp around Scotland (mostly *Laminaria hyperborea*) was estimated as 10 million tons over an area of 8000 km^2^ (Walker 1953). This figure was a map-based estimate derived from detailed surveys of the coastline (Walker and Richardson 1955) over the period 1946–1955, which included aerial photography and quadrat sampling over an area of 270 km^2^ (Walker and Richardson 1956). Interestingly, the resultant time series depicted high interannual variability in kelp biomass in Scotland, which, at the time, was attributed to an 11-year cycle in sunspot activity (Walker 1956). However, re-examination of the data suggests that the highest annual biomass estimates were recorded in years following North Atlantic Oscillation (NAO)-positive summers (Folland et al. 2009). As such, it could be that calm, sunny weather led to increased biomass, suggesting that decadal and shorter term NAO variation may be linked to kelp productivity.

Technological advances in scuba diving in the 1960s and 1970s facilitated stepwise progress in our understanding of the distribution and ecology of kelp forests in the UK. Perhaps, most notable were the seminal body of work by Joanna Kain on the ecology of *Laminaria* on the Isle of Man (see Kain 1979; for overview) and P.G. Moore's work on faunal assemblages within kelp holdfasts in NE England (Moore 1971, 1973). Moreover, between 1970 and 2000, substantial survey work was conducted by the Nature Conservancy Council (NCC) and various successor bodies including the Marine Nature Conservation Review (MNCR). During this time, scuba divers conducted semiquantitative surveys along the majority of the subtidal rocky coastline of the UK, to benchmark patterns of marine biodiversity. This dataset is freely available through the National Biodiversity Network Gateway and remains the only large-scale, systematic assessment of subtidal rocky reef assemblages in the UK.

**Figure 1:**
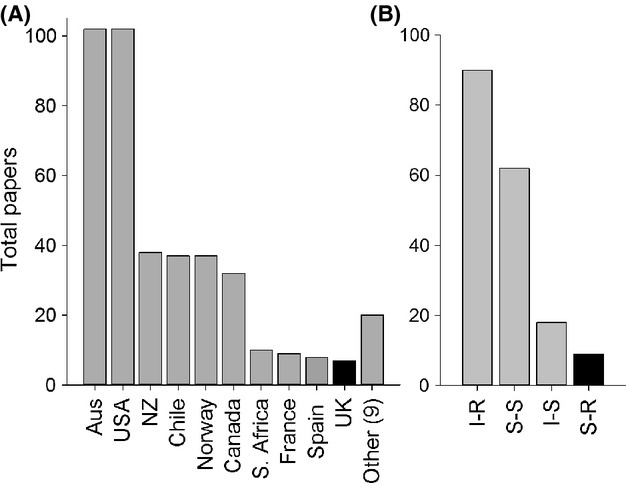
(A) Number of kelp ecology papers by nation (ISI Web of Science search on “kelp,” 2002–2011, *n* = 402 ecology papers). (B) Number of ecology papers focussed on each major benthic marine habitat type in the UK (2002–2011, *n* = 187 papers); I-R = intertidal rocky, S-S = subtidal soft, I-S = intertidal soft, S-R = subtidal rocky.

From the 1980s onwards, changes in attitudes and regulations concerning scientific scuba diving, coupled with shifts in research priorities, and relatively little commercial interest in kelps, have led to a dearth of primary research on kelp forests in UK waters. Subtidal kelp forests persist along >12,000 miles of UK coastline, yet the volume of directed research in recent years pales in significance when compared with kelp studies conducted in other research-intensive nations (Fig. 1). For example, an ISI-listed search of “kelp” papers showed that researchers in Australia and the USA published >100 papers on the ecophysiology or ecology of kelps in the last decade, whereas just seven papers originated from the UK (Fig. 1). Indeed, in the period 2002–2011, more kelp ecology papers originated from sub-Antarctic regions than from the UK. Similarly, a search of marine ecology papers focussing on major habitat types in the UK over the same time frame shows that compared with work on subtidal rocky reefs, 10 times as much research was conducted on intertidal rocky shores; seven times as much, on subtidal soft sediments; and twice as much, on intertidal soft sediments (Fig. 1). With the notable exception of Norwegian research, kelp ecosystems in the wider NE Atlantic have been relatively understudied in recent years (Fig. 1). As the structure of and current threats to kelp forests off Norway are dissimilar to those further south, generalizing the ecological patterns, processes, and predictions to the wider NE Atlantic is problematic. Clearly, the lack of focussed process-based research over recent years has resulted in significant knowledge gaps concerning the responses of kelp-dominated habitats to environmental change, the contribution of kelps and their associated biodiversity to marine food webs, and the resilience of kelp communities to perturbation.

## Kelp Forest Structure

**Figure 2:**
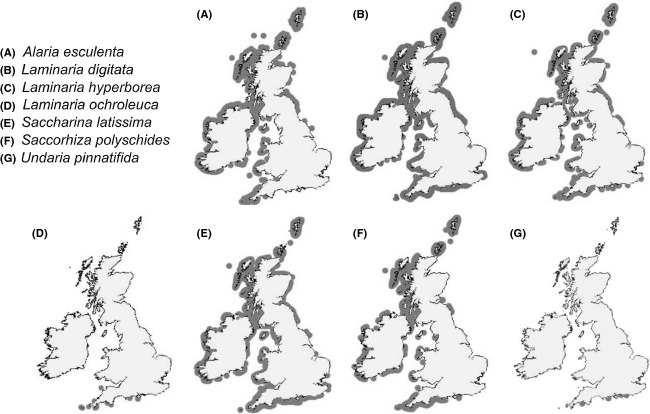
Dark gray hatching indicates the recorded distributions of kelp species in the UK and Ireland (data reproduced from MarLIN, with permission).

In the NE Atlantic, kelps occupy subtidal rocky reefs in all but the most sheltered or turbid locations. Dense kelp forests are found from the lower shore to depths >20 m, from northern Norway and Iceland through to Portugal and Morocco (Hiscock 1998; Bolton 2010). Dominant canopy formers are generally (but not always) members of the family Laminariaceae (e.g., *L. hyperborea, Laminaria digitata, Laminaria ochroleuca*), which exhibit an alternation of dissimilar generations; an asexual diploid phase (the sporophyte) that is usually of considerable size and a haploid dioecious phase (the gametophyte) that is microscopic (Kain 1979). Sporophytes of members of the Laminariaceae comprise a holdfast, a stipe, and a blade, which may comprise many digitate fronds as in *L. hyperborea* or a single undivided frond as in *Saccharina latissima*. In the UK and Ireland, suitable rocky reef habitat is found along much of the undulating coastline, particularly along the wave-exposed south, west, and north coasts. As such, kelps occupy rocky reefs and artificial hard structures from the low water mark to, in extreme cases, depths in excess of 40 m (e.g., *Alaria esculenta* off Rockall, Scotland) along most of the coastline of UK and Ireland (Fig. 2). Kelp forests in the region are complex, as seven different kelp species co-exist, of which 4 are long-lived climax canopy-forming species (Table 1), and their relative abundance is influenced by a range of abiotic (e.g., temperature, latitude, wave exposure, light levels, disturbance) and biotic (e.g., competition, grazing) factors. Even so, the dominant canopy former on most subtidal reefs is *L. hyperborea*, which is a “stipitate” kelp species with a rigid stipe (1–3 m long) that holds the fronds above the substratum. *Laminaria hyperborea* is distributed from the Arctic south to northern Portugal, and in the UK, it persists on all but the most wave-exposed or turbid rocky reefs. The sporophyte becomes fertile after 2–6 years and may live for 5–18 years in the UK (Kain 1979). *Laminaria hyperborea* influences its environment and other organisms by providing food and habitat and by altering light, water motion, sediment deposition, and physical disturbance through thallus scour. It is, in the truest sense, an ecosystem engineer and functions as the assemblage dominant by outcompeting other large macroalgae under most conditions (Hawkins and Harkin 1985).

**Table 1:** Kelp species in UK and Irish waters. The geographic range and approximate depth range, typical mature sporophyte length, and lifespan of kelps in UK/Irish waters are shown. Also shown is the predicted change in abundance and/or range of each species in response to continued environmental change.

Species	Distribution	Depth range (m)	Length (m)	Lifespan (years)	Change (?)
*Laminaria hyperborea*	Arctic–Portugal	0–30	1–3	5–18	Decrease
*Laminaria digitata*	Arctic–France	0–15	1–2	4–6	Decrease
*Laminaria ochroleuca*	UK–Morocco	0–30	1–3	5–18^1^	Increase
*Saccharina latissima*	Arctic–France	0–30	1–3	2–4	Decrease
*Alaria esculenta*	Arctic–France	0–35	1–2	4–7	Decrease
*Saccorhiza polyschides*^2^	Norway–Morocco	0–35	2–3	1	Increase
*Undaria pinnatifida*	Global NIS^3^	0–15	1–3	1	Increase

1 The lifespan of *L. ochroleuca* in UK waters is unknown and is estimated based on its close affinity with *Laminaria hyperborea*.

2 *S. polyschides* is not a true kelp of the order Laminariales (being of the order Tilopteridales), but is included as this “pseudokelp” can perform a similar ecological role as the dominant canopy former.

3 *U. pinnatifida* is a nonindigenous species (NIS) within the NE Atlantic, having originated from the NW Pacific.

Other members of the genus found in UK waters are *L. digitata* and *L. ochroleuca*. *Laminaria digitata* is distributed from Arctic waters to its southern range edge in Brittany, France. It is perennial, reaching maturity after 1–2 years and persisting for up to 6 years and is smaller than *L. hyperborea*, reaching a maximum total length of 3 m. *Laminaria digitata* tends to dominate the low intertidal and immediate subtidal zones, but is outcompeted by *L. hyperborea* at depths of a few meters (Kain 1975a; Hawkins and Harkin 1985). In contrast to *L. hyperborea*, its stipe is very flexible so that fronds scour the immediate substratum, which facilitates attachment in the wave-exposed shallow subtidal zone. *Laminaria ochroleuca* is a warm-temperate Lusitanian species, which is distributed from the south of England to Morocco and occurs in both the Straits of Messina and the Azores. It is very similar in morphology to *L. hyperborea* and is thought to serve a similar ecological function, although little is known about its ecology in UK waters (Blight and Thompson 2008). *Laminaria ochroleuca* is thought to be expanding its range polewards, perhaps in response to ocean warming. It was first recorded in the far southwest of England in 1948 and has subsequently progressed eastwards as far as the Isle of Wight and northwards onto Lundy Island in the Bristol Channel (Blight and Thompson 2008; Brodie et al. 2009). Long-established populations on the south coast are also thought to be increasing in abundance, perhaps at the expense of *L. hyperborea* (K. Hiscock, pers. comm.).

The remaining kelp species are structurally and functionally diverse and can be locally abundant and sometimes dominant. *Saccharina latissima (*formerly *Laminaria saccharina)* has a short stipe and a single, undivided frond (up to 4 m in length) with a “frilly” undulating margin. It is a short-lived perennial, reaching maturity at 1–2 years and living for up to 4 years. *Saccharina latissima* is found from the Arctic to France (although some isolated populations in northern Portugal may persist) and tends to attach to semistable substrata (e.g., boulders) or inhabit the margins of dense *L. hyperborea* forests, particularly in sheltered to moderately exposed locations. In sheltered embayments, where sedimentation is high and wave action is low (such as in Scottish sea lochs), *S. latissima* is often the assemblage dominant. *Alaria esculenta* has a similar distribution and, in many respects, morphology (having a short stipe and single blade with distinct midrib extending to 1–3 m in length), but is restricted to wave-exposed conditions and attaches to stable substrata. It is fertile in about 1 year and lives for 4–7 years. *Alaria esculenta* mostly functions as a midsuccessional species and is outcompeted by members of the genus *Laminaria*, except under extremely wave-exposed conditions where it may dominate the assemblage (Hawkins and Harkin 1985). Finally, two short-lived, annual kelp species are found in waters off the UK and Ireland: *Saccorhiza polyschides* and the non-native *Undaria pinnatifida* (“Wakame”). *Saccorhiza polyschides* is not a “true kelp” of the order Laminariales, being a “pseudo-kelp” of the order Tilopteridales (see Sasaki et al. 2001 and references therein), but is treated as a kelp here because it serves a similar ecological function and can be the dominant canopy-forming macroalgae along large stretches of the NE Atlantic coastline. *Saccorhiza polyschides* is found from Norway to Morocco and can be the dominant canopy former in warmer waters where *L. digitata* and *L. hyperborea* are absent (Hawkins and Harkin 1985). It is particularly abundant off the southwest coast of Ireland and common throughout much of the UK (Norton 1978). It is a fast-growing opportunistic species that can tolerate very calm through to very turbulent conditions, attaches to a range of substratum types, and is often found at the margins of dense *Laminaria* forests (Norton 1969). There has been some evidence to suggest that the relative abundance of *S. polyschides* has increased along the south coast of England (Birchenough and Bremmer 2010; S. J. Hawkins, pers. obs.), but reliable data are lacking. There is little doubt, however, that the abundance and distribution of the global invader *U. pinnatifida* have increased in UK waters in recent decades; having first been recorded on the south coast of England in 1994 (Fletcher and Manfredi 1995), it has now become established at a number of locations in the UK (Farrell and Fletcher 2006). *Undaria pinnatifida* can tolerate a wide range of salinities, temperatures, and sediment loads and, as such, has become abundant in many marinas, estuaries, and embayments in Europe (Castric-Fey et al. 1993; Fletcher and Manfredi 1995). In Plymouth Sound (UK), for example, *U. pinnatifida* is now the dominant macrophyte on both natural and artificial substrata throughout spring and early summer (D. A. Smale, unpubl. obs.).

The structure of entire kelp forests – in terms of the identity and abundance of kelp species and their associated biodiversity – varies considerably in space and time as a function of wave exposure (and storm frequency and magnitude), light levels (influenced by depth and turbidity), sedimentation, and temperature. As a general rule, in moderately exposed conditions, dense stands of *L. digitata* will persist from the low water mark to a few meters depth, with the upper limit of *L. digitata* set by physical stress and competition with *Fucus serratus* (Hawkins and Harkin 1985) and the lower limit set by competition with *L. hyperborea*, which is mediated by wave exposure (Kain 1962; Hawkins and Harkin 1985). *Saccharina latissima* and *S. polyschides* generally inhabit the immediate subtidal, fringes of rocky reefs, or boulders (Kain 1962). As the substratum extends into deeper water and light becomes limiting, the density of kelps decreases, and isolated (often large) individuals of *L. hyperborea* and *S. polyschides* replace dense stands. In some locations, such as off the Isle of Man (UK) and in Lough Hyne (Ireland), grazing by sea urchins may control the lower depth limit of kelp forests (Kitching and Ebling 1961; Jones and Kain 1967; Kain 1975a). While many kelp-dominated systems are dynamic and exhibit pronounced spatiotemporal variability at multiple scales (see Wernberg and Goldberg 2008; Smale et al. 2010 for Australian examples), others are relatively more stable. For example, southerly distributed European kelp forests (i.e., along the Iberian Peninsula) are more prone to short-term temporal variability arising from variations in both the strength of coastal upwelling and recruitment patterns of dominant canopy formers (e.g., Tuya et al. 2012). Similarly, high-latitude kelp forests may exhibit considerable temporal variability over years to decades, driven by stochastic (or perhaps cyclical) periods of overgrazing by sea urchins, in particular *Strongylocentrotus droebachiensis* (Norderhaug and Christie 2009). It could be that midlatitude kelp forests are more stable within ecological timescales, although explicit comparisons of variability patterns along broad-scale latitudinal gradients are lacking.

**Figure 3:**
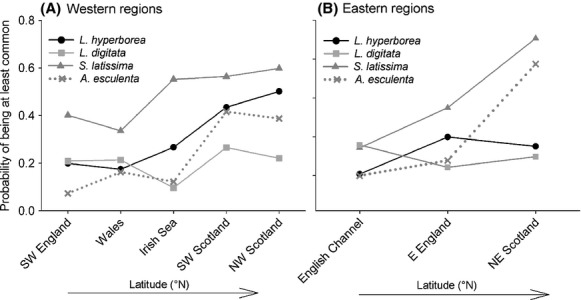
Habitat-specific probability of occurrence for dominant kelp species in UK waters, for both western regions (A) and eastern regions (B), along a latitudinal gradient (∼49–59°N). Probabilities derived from subtidal habitat surveys conducted at 0–10 m depth (data from Marine Nature Conservation Review, 1977–2000, see Burrows 2012 for more methodological details and geographic limits of regions), which used ACFOR values (a semiquantitative abundance scale) to quantify benthic organisms. The number of independent surveys per region (i.e., *n*) ranged from 300 to 734.

At regional spatial scales across the UK and Ireland, there are some general trends in kelp forest structure that are primarily driven by the abundance distribution patterns of individual kelp species. The occurrence of the cold water kelps *L. hyperborea*, *S. latissima*, and *A. esculenta* generally increases with latitude from southern England to northernmost Scotland (Fig. 3), which corresponds to a geographic shift from the southern limit toward the center of these species’ distributions. Broadly speaking, optimal kelp habitat off the west and north coasts of Scotland is characterized by dense stands of *L. hyperborea* (wave-exposed) or *S. latissima* (more sheltered), whereas kelp forests off the south and west coasts of the UK and Ireland are more mixed, with a greater relative abundance of *S. polyschides* and *L. ochroleuca*. This regional-scale shift in kelp forest structure occurs over a latitudinal temperature gradient of some 3°C and may provide some insights into the likely effects of gradual seawater warming on kelp forest structure and function (see “Climate change” section).

## Ecological Goods and Services

**Figure 4:**
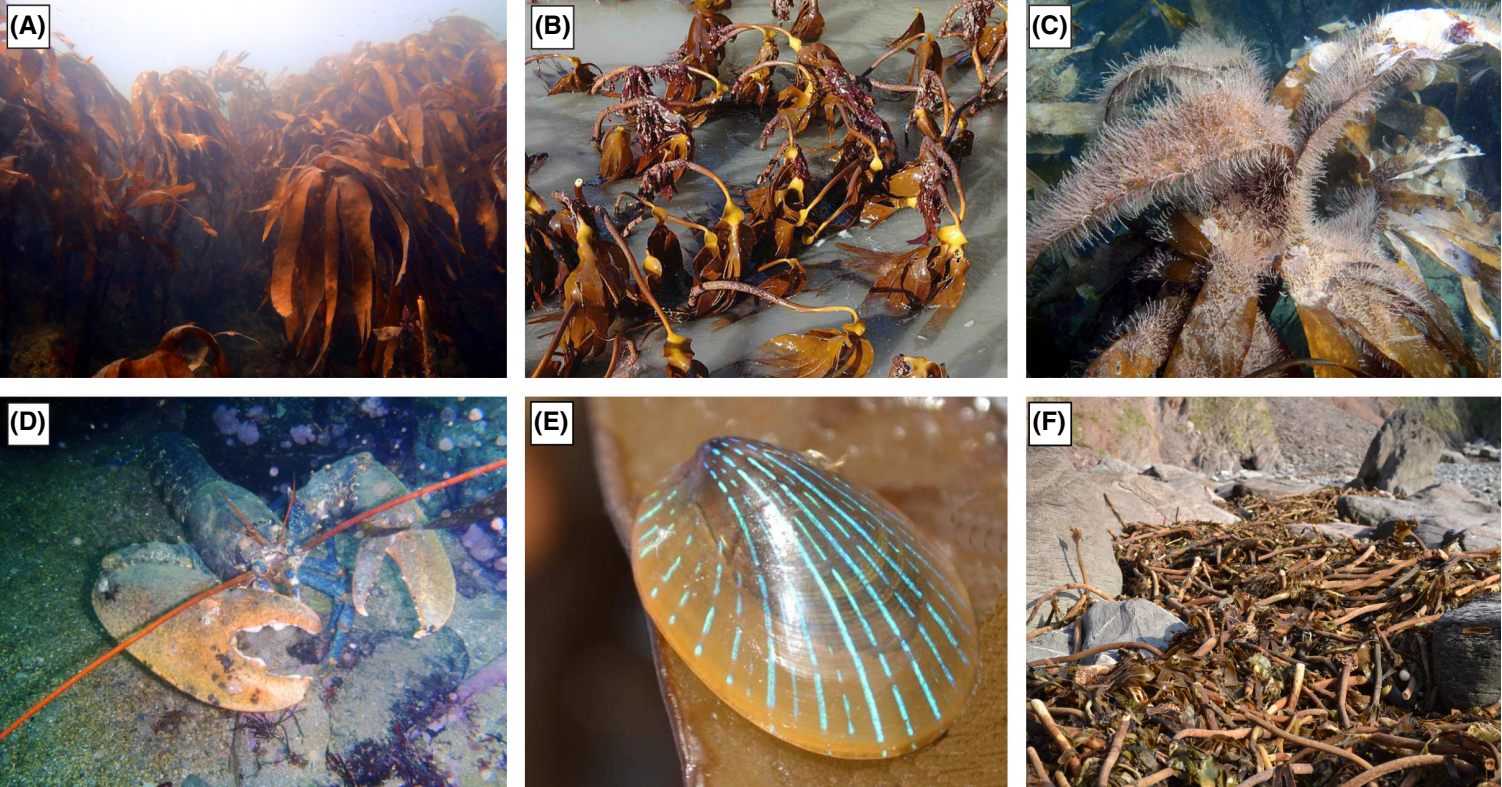
The kelp *Laminaria hyperborea* is a dominant canopy former on both subtidal (A) and intertidal (B) rocky reefs around the UK and the wider NE Atlantic. Kelp forests provide habitat for a wide range of flora and fauna, including the hydroid *Obelia geniculata* (C) and the commercially important European Lobster *Homarus gammarus* (D). Although kelps and their epiphytes are grazed directly, by the blue-rayed limpet *Patella pellucida* for example (E), the majority of kelp production is consumed as detritus (F).

Kelps are hugely important as primary producers (both locally and via export of detritus to nearby habitats), as habitats and repositories of marine biodiversity and secondary productivity, as natural coastal defense, and as nursery grounds for exploited species (reviewed by Steneck et al. 2002). Specific UK-based examples of these roles are illustrated in Figure 4 and described in detail below.

### Biodiversity

Habitat-forming species or “engineers” (sensu Jones et al. 1994), such as kelps and corals, exert control over entire communities by modifying the environment and resources available to other organisms (e.g., Bertness and Callaway 1994; Jones et al. 1997). In particular, kelps alter light (Wernberg et al. 2005), sediments (Wernberg et al. 2005), physical scour (Konar and Estes 2003), and water flow (Stewart et al. 2009) for proximal organisms while providing structural habitat for a wide range of flora and fauna. Within the UK alone, more than 1800 species of flora and fauna have been recorded from kelp-dominated habitats (MNCR, unpubl. data). As habitat formers, a single kelp directly provides three distinct primary habitats; the holdfast, the stipe, and the lamina. In addition, epiphytes (primarily attached to the stipe) provide a secondary habitat for colonization. Over 40 years of descriptive research on kelp-associated faunal assemblages in the NE Atlantic has unequivocally demonstrated that kelps harbor considerable biodiversity (e.g., Moore 1971, 1973; Edwards 1980; Christie et al. 2003; Blight and Thompson 2008). For example, a study on *L. hyperborea* in Norway by Christie et al. (2003) showed that on average, a single kelp plant supports ∼40 macroinvertebrate species represented by almost 8000 individuals. The biogenic habitat formed within the kelp holdfast generally harbors the most diverse assemblages, with species richness per holdfast typically in the region of 30–70 macrofaunal species (Edwards 1980; Christie et al. 2003; Blight and Thompson 2008). However, assemblage richness and structure are strongly influenced by the volume and complexity of the holdfast habitat (e.g., Blight and Thompson 2008), as well as by external local and regional factors (e.g., turbidity, exposure). The secondary habitat formed by epiphytes on kelp stipes is often utilized by a highly abundant and diverse fauna (Christie et al. 2003), which varies considerably in space (i.e., with location and depth) and time (i.e., with season and year). Kelp lamina generally supports lower diversity, although epiphyte growth can be very extensive under certain conditions. While diversity may be low, the abundance of several widespread epibionts of kelp lamina (e.g., the blue-rayed limpet, *Patella pellucida*, Fig. 4 and the “sea mat” bryozoan *Membranipora membranacea*) can be locally very high (Christie et al. 2003). Kelps facilitate other species by initiating a “habitat cascade” (Thomsen et al. 2010), in which kelps provide habitat for other sessile flora and fauna, which in turn support a wide array of mobile invertebrates.

At spatial scales larger than that of a single kelp plant, multiple individuals form extensive forests that provide three-dimensional habitat for a vast array of marine organisms. Rich understorey assemblages of plants and animals persist beneath kelp canopies, which ameliorate environmental stressors, and provide shelter and food. With respect to understorey macroalgae, more than 40 species (principally rhodophytes) are regularly found beneath kelp canopies (Maggs 1986), although their relative abundance varies considerably between biogeographic regions and is strongly influenced by local factors such as depth, turbidity, wave exposure, and siltation (Maggs 1986). Studies in other temperate regions have indicated that diverse macroalgal canopies may support greater biodiversity in understory assemblages compared with mono-specific canopy stands (Smale 2010), perhaps because structurally varying canopy formers enhance habitat diversification. While this has not yet been examined in UK waters, the region represents a tractable model system due to the co-existence of several canopy-forming kelp species.

**Figure 5:**
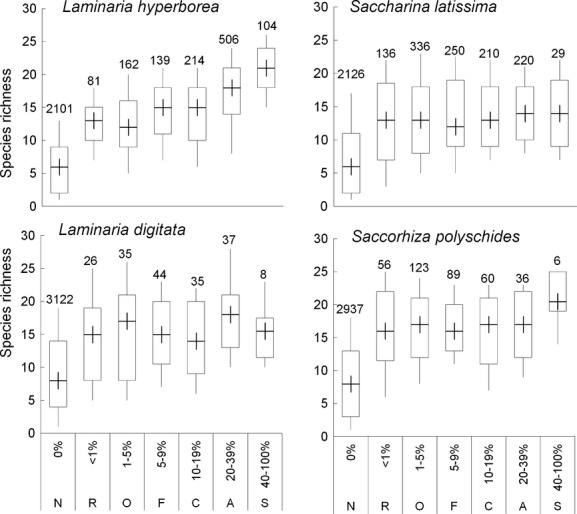
Kelp species abundance and local species richness. Box plots show 10th, 25th, 50th, 75th, and 90th percentiles of species richness data for each modified SACFOR category of kelp species abundance. For each SACFOR category, *n,* which is the number of independent surveys conducted during the Marine Nature Conservation Review (1977–2000), is given.

Kelp forests in the UK and Ireland also provide habitat for large invertebrates, such as gastropod molluscs, crustaceans, and echinoderms, some of which have significant ecological (e.g., sea urchins, see Jones and Kain 1967; Kitching and Thain 1983) or socioeconomic (e.g., the European lobster, see Johnson and Hart 2001) importance. Kelp forests are particularly effective nurseries for juvenile invertebrates and fish (e.g., Atlantic cod and pollock), which provide shelter from predation. Moreover, kelp forests are key feeding grounds for many NE Atlantic fish species, such as *Labrus bergylta* (ballan wrasse) and *Ctenolabrus rupestris* (goldsinny wrasse), which prey on kelp-associated invertebrates (Norderhaug et al. 2005). In turn, elevated fish densities in kelp forests attract large piscivores, such as large fish, seals, and otters. In general, subtidal rocky reefs with extensive stands of *L. hyperborea* support greater species richness than reefs without high kelp coverage (Burrows 2012). Further analysis indicates that species richness on subtidal rocky reefs around the UK generally increases with increasing relative abundances of all the major canopy-forming kelp species (Fig. 5).

The vast majority of work on kelps as habitat formers and repositories of biodiversity has focussed on *L. hyperborea*. What is clear, however, is that different kelp species have different morphologies and life histories and, as such, provide structurally varying habitat. This is important within the context of environmental change, as any shifts in the relative abundance of kelp species may have knock-on effects on their associated biodiversity. For example, understorey assemblages associated with *L. digitata* are distinct from those beneath *L. hyperborea* because the stipe of the former is shorter and less rigid. As a result, the substrate near *L. digitata* plants experiences greater physical abrasion by lamina such that fewer species can inhabit the understorey compared with *L. hyperborea* (Kain 1979). However, certain species such as the limpet *Patella ulyssiponensis* and the sponge *Halichondria panicea* are facilitated by “sweeping” by *L. digitata,* as they would otherwise be outcompeted by understorey algae. Similarly, subtle differences in morphology (e.g., holdfast volume and complexity, stipe roughness, and susceptibility to epiphyte growth) can have a strong influence on the structure and richness of associated assemblages (e.g., Blight and Thompson 2008). The nature of interspecific and regional-scale variability in kelps as habitat formers within the UK and Ireland (and the wider implications for biodiversity) is poorly understood and remains an important knowledge gap within the field of kelp forest ecology.

### Productivity and food webs

Kelp forests represent some of the most productive habitats on the Earth (Mann 1973, 2000; Reed et al. 2008) and are a major source of primary production in coastal zones of temperate and polar oceans worldwide (Steneck et al. 2002). Kelp productivity is strongly correlated with nutrient availability (Gagné et al. 1982), but is also affected by temperature (Bearham et al. 2013), wave exposure (Pedersen et al. 2012), light (Bearham et al. 2013), and disturbance regime (Reed et al. 2008). Moreover, kelp populations have the potential to acclimatize or adapt to local conditions to maintain productivity rates (Gagné et al. 1982; Delebecq et al. 2013). Extension (i.e., growth) rates of kelp vary considerably between geographic regions and between species, as they are closely related to morphology and growth strategy. Even so, when growth rates are converted to biomass production per unit area different kelp species tend to exhibit broadly comparable productivity rates (Mann 1973; Fairhead and Cheshire 2004; Krumhansl and Scheibling 2012).

In the Atlantic, kelp primary production can be in excess of 1000 g C m^−2^·year^−1^ and that from *Laminaria* species has been estimated at between 110 and 1780 g C m^−2^·year^−1^ (Mann 1973, 2000), while primary production from phytoplankton in coastal temperate regions is typically between 100 and 300 g C m^−2^·year^−1^ (Mann 2000). Given these relative rates of production, it is possible to approximate the relative proportion of primary production derived from both phytoplankton and benthic macroalgae in UK coastal waters. Walker (1953) estimated an area of 8000·km^−2^ of kelp habitat in Scotland alone, which may produce 10 M t C·year^−1^ at typical production rates of 1300 g C m^−2^·year^−1^ (Dayton 1985). This compares with a potential phytoplankton production of 13 M t C·year^−1^ from 133,000 km^2^ of sea <20 km from the coast within the UK exclusive economic zone (EEZ) and 73 M t C·year^−1^ from the 770,000 km^2^ of the entire UK EEZ (assuming a rate of production from phytoplankton of 100 g C m^−2^·year^−1^). Therefore, kelp may conservatively account for ∼45% of primary production in UK coastal waters, and 12% of marine production in the entire UK EEZ. This estimate for annual UK kelp production does not include the extensive shallow subtidal rocky reef habitats found off England and Wales and will therefore be an underestimate. Moreover, when primary productivity rates of intertidal macroalgae are compared with subtidal macroalgae, intertidal production is typically 10% of that from the subtidal (Mann 2000). Although these coarse estimates should be interpreted with caution, it is clear that kelps make a substantial contribution to primary production in coastal waters off the UK and Ireland.

Some kelp biomass is consumed directly by herbivorous fish and invertebrates, such as the conspicuous blue-rayed limpet *P. pellucida* (Fig. 4). However, >80% of kelp production enters the carbon cycle as detritus or dissolved organic matter, because little is directly grazed by herbivores (Krumhansl and Scheibling 2012). Kelps act as “conveyor belts” of biomass production, as the meristematic tissue is (generally) located at the junction between the stipe and the lamina so older tissue is passed distally with continued growth. At the distal end of the blade, tissue is rapidly or gradually eroded to generate detrital fragments ranging in size from small particulates to large sections of blade. As kelp blades fragment, dissolved organic matter is released, which may account for up to 35% of annual energy production (see Krumhansl and Scheibling 2012 and references therein). During times of high water motion (i.e., during intense storms or at highly exposed locations), whole kelps may be dislodged following detachment at the holdfast or breakage at the stipe. The proportion of kelp production that is either eroded as fragments or dislodged as whole plants varies among species and with morphology and age of kelp. De Bettignies et al. (2013) recently showed that erosion of the kelp *Ecklonia radiata* accounted for ∼80% of detritus production, with dislodgement comparatively less important. Similarly, it is thought that erosion rates generally exceed dislodgement rates for *Laminaria* and *Saccharina* spp., although direct comparisons are lacking (Krumhansl and Scheibling 2011).

Kelp detritus is either retained within the kelp forest or exported to adjacent habitats by water movement driven by currents, tides, or waves. Rates of export exhibit pronounced spatiotemporal variability as they are governed by a complex, interacting suite of factors including water flow, seabed topography, substratum type, and aspects of the detritus itself (e.g., size, buoyancy, density, and age). Kelp detritus may settle locally and form a food source for a wide range of benthic invertebrates (Duggins and Estes 1989; Norderhaug et al. 2003), or be transported to adjacent (Tallis 2009) or distant habitats (Vanderklift and Wernberg 2008). Either way, most kelp-derived carbon is consumed by suspension feeders, detrital grazers (such as limpets and *Littorina littorea*), and general consumers of organic material in soft sediments (deposit feeders). An important, but poorly understood, process relating to kelp detritus consumption concerns the interactions between microbes and macrofauna. It is clear that microbial degradation of kelp tissue increases palatability for many grazers by reducing C:N ratios and phlorotannin content (Norderhaug et al. 2003), but the influence of microbial processes on palatability varies between species of kelps (Duggins and Eckman 1997) and grazers (Norderhaug et al. 2003), and microbial degradation may be less important than for angiosperms such as sea grasses (Bedford and Moore 1984).

Kelp detritus is particularly important as a spatial subsidy of energy into low-productivity habitats, the most visible example being the deposition of kelp wrack into sandy beach habitats, where it provides a principal food sources for rich and abundant microbial and faunal assemblages (Ince et al. 2007). Similarly, exported kelp represents a spatial energy subsidy into sea grass meadows (Wernberg et al. 2006; Hyndes et al. 2012), soft sediments (Bedford and Moore 1984; Vetter and Dayton 1998), subtidal reefs (Vanderklift and Wernberg 2008), and rocky intertidal habitats (Bustamante and Branch 1996; Tallis 2009). Kelp detritus may be consumed many kilometers from its source (Vanderklift and Wernberg 2008) and, following offshore transportation, may enrich soft sediments at depths of 900 m or more (Vetter and Dayton 1998). In the UK and Ireland, targeted research on kelps as fuels of coastal food webs has been lacking, and specific rates of kelp detritus production and export remain almost entirely unknown (but see Johnston et al. 1977 for experiment on *S. latissima* in Scotland). Evidence from elsewhere would indicate that kelp biomass is a hugely important source of exported energy, which influences patterns of secondary production and the distributions of marine organisms. Detritus production and export rates are likely to vary considerably between regions and seasons, and the quantity and quality of exported material will vary between kelp species. Using evidence from data-rich systems (e.g., northwest Atlantic) will facilitate the formation of testable hypotheses that can direct field-based research needed to enhance understanding of trophic processes and, ultimately, support management decisions.

The fraction of carbon fixed by kelps that is effectively removed from the atmosphere over decadal to century timescales is as yet poorly understood. The process of incorporation into longer term stores of carbon may depend on the export of particulate kelp detritus from coastal habitats into sediment in deeper water or the export of recalcitrant dissolved carbon into deep ocean water, but the potential for such storage (and thereby influence on the carbon budget) is not inconsiderable.

### Coastal defense

Kelp forests, such as other biogenic structures in coastal zones (e.g., salt marshes, mangroves), prevent and alleviate the damage caused by flooding and storm events. Kelps forests alter water motion and provide a buffer against storm surges through wave damping and attenuation and by reducing the velocity of breaking waves (Lovas and Torum 2001). In doing so, kelp forests reduce coastal erosion and the movement of sand and pebbles from adjacent beaches (Mork 1996; Lovas and Torum 2001). However, compared with other coastal habitat formers (e.g., mangroves, corals), there is a paucity of information on the degree of storm protection offered by kelp forests. It is clear that the magnitude of wave damping is strongly influenced by the morphology and drag co-efficient of the dominant kelp species and, as such, will vary between biogeographic regions. Moreover, the degree of water flow attenuation by kelp forests is correlated with the extent, density, and morphology of both the canopy-forming kelps (Gaylord et al. 2007) and the understorey macroalgal assemblage (Eckman et al. 1989). Other studies on various submerged vegetation types have also found significant relationships between the extent of vegetation and the degree of wave damping and coastal erosion (e.g., Türker et al. 2006). Off Norway, *L. hyperborea* forests may reduce wave heights by as much as 60% (Mork 1996). As such, *Laminaria* forests in the UK and Ireland may similarly offer some degree of coastal defense and are probably locally important to some coastal settlements. Coastal defense represents a critical ecosystem service that will become more important along many coastlines as the consequences of anthropogenic climate change intensify, namely sea-level rise and an increased magnitude and frequency of storms.

### Goods

Living resources derived from kelp-dominated habitats have long been exploited by humans. Indeed, the recently proposed “kelp highway” hypothesis suggests that kelp forests may have facilitated the movement of maritime peoples from Asia to America some 16,000 years ago. Around this time, a deglaciated coastal migration route through the North Pacific – a linear band of highly productive kelp forests extending discontinuously from Japan to Baja California – was probably used by maritime hunter gatherers that subsisted on shelled invertebrates, fish, and large mammals inhabiting kelp habitats (Erlandson et al. 2007). Extensive kelp forests would also have buffered wave energy, offered secure moorings for boats and assisted with navigation and therefore facilitated a coastal, migratory existence (Erlandson et al. 2007). To this day, the magnified secondary productivity characteristic of kelp forest habitats is exploited for human consumption. Previous work in North America has demonstrated that the American lobster (*Homarus americanus*) is affiliated with kelp forests and will preferentially aggregate under *Laminaria* canopies (Bologna and Steneck 1993). In the NE Atlantic, kelp forest habitats are vital for the European lobster, *Homarus gammarus*, where it preys on a variety of molluscs and crustaceans, and are also home to velvet swimming crabs (*Necora puber*) and seasonal spider crab migrants (*Maja brachydactyla*). The lobster fishery is worth ∼£30 m per year to the UK economy alone, while the smaller crab fisheries are important for both export and recreation (Elliott et al. 2012). Kelp forests also serve as a nursery for many fish species, including Atlantic Cod (*Gadus morhua*), and attract commercially important species such as European sea bass (*Dicentrarchus labrax*), pollack (*Pollachius pollachius*), and conger eels (*Conger conger*).

Kelp itself has myriad of uses and applications. The first use of kelps and other macroalgae to feed domestic animals may have occurred as early as the fifth millennia BC, soon after the arrival of the first domestic herds (Balasse et al. 2005). Most famously, a breed of sheep on North Ronaldsay (Orkney Islands, Scotland) feeds almost entirely on beach wrack (principally *L. hyperborea*) for most of the year. Stable isotope analysis suggests that the North Ronaldsay breed has been consuming kelp since the fourth millennia BC, during which time it has adapted its rumen bacteria to facilitate the breakdown of laminarin (the storage glucan in brown algae) and adapted an unusual pattern of grazing and ruminating that follows the tidal cycle rather than the (more typical) diurnal cycle (Balasse et al. 2005). More sophisticated methods are now used to process kelp for animal feed supplements for both agriculture and aquaculture. Kelp is rich in nutrients and alginates, which condition soils, and as such has also long been collected and used as a fertilizer (a practice that is still commonplace in parts of Scotland, Ireland, and the Channel Islands).

Industrial-scale kelp harvesting in Scotland and Ireland stems back to the 17th Century, when it was collected in great quantities and burnt in kelp kilns to produce sodium carbonate (Forsythe 2006). “Kelp ash” was used in the manufacture of glass and soap and for pottery glazing, as well as for fertilizer. Since the early 20th Century, kelps have principally been harvested for alginates, which are used in foods, textiles, and pharmaceuticals. Alginates are extracted chemically and used in bulking, gelling, and stabilizing processes; about 25,000 tonnes of alginate per year is extracted worldwide (Bixler and Porse 2011). Kelp is currently commercially harvested in the northern and western isles of Scotland, while commercial farming of *L. digitata* has recently been developed off the west coast of Ireland. However, the magnitude of kelp harvesting in the UK and Ireland is low in comparison with neighboring France and Norway, where 50,000 tonnes of *L. digitata* and 200,000 tonnes of *L. hyperborea*, respectively, are harvested each year (primarily for alginate production).

The current demand for clean, non-fossil-fuel-based energy production has thrown kelps into the limelight as potential sources of biofuels. Kelps can grow very quickly (up to 50 cm per day), are rich in polysaccharides, and do not compete with land-based crops for space, fertilizers, and water. Moreover, recent advances in bioengineering now allow alginate polysaccharides to be degraded, metabolized, and converted to ethanol (Wargacki et al. 2012). There is therefore increasing global interest in large-scale harvesting and culturing of kelps for biofuels. In Ireland, for example, the EnAlgae project (http://www.enalgae.eu) is cultivating macroalgae in and around Strangford Lough for biofuel development, and similar projects are underway in Scotland. A recent cradle-to-grave analysis of the carbon footprint of the production of biofuels (ethanol and methane) from seaweeds, however, indicated that production of biofuels from other sources (e.g., corn, wheat, sugar cane) is more efficient (Fry et al. 2012). Clearly, the magnitude of kelp production for biofuels would need to be substantial to have any bearing on the energy market, which could have wide-ranging implications for coastal ecosystems that remain poorly understood (see “Threats and Knowledge Gaps” section).

Kelp itself has long been directly consumed by humans. In Asian cuisine, kelps such as *Saccharina japonica* (“Kombu”) and *U. pinnatifida* (“Wakame”) – now a global invasive pest – have been vital ingredients for many centuries (Jaspars and Folmer 2013). In coastal communities in the UK, nonkelp seaweeds have been consumed for at least 4000 years, particularly *Palmaria palmata* (“Dulse”), *Chondrus crispus* (“Carageen”), *Porphyra umbilicalis* (“Purple laver”), and *Ulva lactuca* (“Green laver”). Although all kelps in the UK and Ireland are edible, *S. latissima* is considered the most palatable due to its sweet taste. Kelp “crunchies” – a cornbread snack flavored with *A. esculenta* – were briefly on the market in the 1980s–1990s, but failed to achieve mainstream popularity. More recently, kelps including *A. esculenta* and *S. latissima* are being marketed as “sea vegetables” by health food companies, due to their high levels of vitamins and minerals and low levels of salt and digestible sugars (Jaspars and Folmer 2013). As such, some suppliers in Scotland and Ireland harvest kelps for human consumption, but these operations are currently fairly small scale.

### Socioeconomic importance

Coastal marine biodiversity in the UK and Ireland is of significant socioeconomic importance. For example, Beaumont et al. (2008) calculated that the leisure and recreation industries directly reliant on coastal marine biodiversity contribute >£11 billion to the UK economy each year. In addition to this monetary value, engagement with marine life has considerable benefits for human health and wellbeing and has directly influenced cultural and economic activities for thousands of years. Kelps as primary producers and habitat providers play a key role in the maintenance of fish stocks and ecosystem structure and therefore indirectly help to sustain regional fisheries and the coastal communities they support (see “Goods” section above for examples). Diverse, healthy kelp-dominated habitats offer a range of recreational activities, which significantly contribute to regional economies and have wider benefits from human health and wellbeing (Beaumont et al. 2008). Key recreational activities associated with kelp forests include snorkeling, scuba diving, free diving, kayaking, wildlife watching, and angling (Beaumont et al. 2008).

In Lyme Bay (a medium-sized embayment off the south coast of England), recreational scuba diving – much of which is conducted on submerged kelp-dominated rocky reefs – contributes >£2.5 million per year to the local economy and supports ∼10 independent dive operators (Rees et al. 2010). With regard to sea fishing, the total expenditure by anglers resident in England and Wales is estimated at £538 million per year from 12.7 million angler days (estimate for 2004, see Beaumont et al. 2008). Although this activity is not wholly focused on or near kelp forests, submerged rocky reefs are often favored by anglers targeting demersal species, and as such, a substantial component of that valuation relies on kelp forest biodiversity. The socioeconomic importance of kelp forest habitats is magnified in isolated coastal regions such as the Western Isles of Scotland and the Isles of Scilly. The vast kelp forests along the north and west coasts of Scotland support abundant wildlife, such as sea birds, seals, and otters, and the value of this biodiversity to local economies through “green” tourism has long been recognized. Similarly, tourism accounts for 85% of the economy of the Isles of Scilly, primarily although coastal-based activities such as sea angling, seal and bird watching, and scuba diving (Beaumont et al. 2007). Much of this is based around the widespread shallow water kelp forests that extend from the islands.

Finally, there are myriad of nonmonetary benefits derived from kelp forest biodiversity. There is growing appreciation for the “feel good” or “warm glow” benefits, which are derived from marine organisms without using them (Beaumont et al. 2007). Kelp-associated species, from seaweeds to sea stars to seals, have inspired artists, facilitated educators, and fascinated tourists for many generations.

## Threats and Knowledge Gaps

### Climate change

In Europe and elsewhere, marine plants and animals have undergone climate-driven shifts in their distributions (Sunday et al. 2012; Poloczanska et al. 2013), and major changes in assemblage structure and ecosystem function are projected to occur as a result (Helmuth et al. 2006; Hawkins et al. 2009). While patterns of ecological change, and the processes driving them, have been well documented in both intertidal (Helmuth et al. 2006; Hawkins et al. 2009) and pelagic (Richardson and Schoeman 2004) systems, there is currently limited information from subtidal benthic systems, especially from hard-bottom habitats that cannot be routinely trawled, dredged, or cored. This was highlighted by the recent “Marine Climate Change Impacts Knowledge Gaps” report, which stated that *knowledge of large scale benthic species distributions within UK waters is required, to detect changes over large areas of the seabed and patterns of benthic response to climate change*. This understanding is urgently needed to maintain *healthy and biologically diverse seas* (MCCIP 2012).

Kelps are cool-water species that are stressed by high temperatures (Steneck et al. 2002), so that seawater warming will affect the distribution, structure, productivity, and resilience of kelp forests (Dayton et al. 1992; Wernberg et al. 2010; Harley et al. 2012). Poleward range contractions have been predicted for several more northerly distributed kelp species (e.g., *A. esculenta*, *L. digitata*, *L. hyperborea*) in response to ocean warming in the Atlantic (Hiscock et al. 2004; Muller et al. 2009; Raybaud et al. 2013). It is evident that the relative abundance of several kelp species changes with latitude along NE Atlantic coastlines, which corresponds to a regional-scale temperature gradient, and that several habitat-forming kelps are at their range edge in the UK and Ireland (e.g., *L. ochroleuca* at its northernmost limit, *A. esculenta* at its southernmost limit, Fig. 3). Because of these distribution patterns and because the distributions of some intertidal species have shifted, several authors have predicted that relatively southerly distributed species will increase in abundance, while more northerly species will decrease in abundance and/or undergo range contractions in the UK and Ireland (Breeman 1990; Hiscock et al. 2004). There is some evidence to suggest that more southerly distributed kelp species (e.g., *L. ochroleuca* and *S. polyschides*) have increased in abundance and have undergone poleward range-edge expansions, while conversely, northern species (e.g., *A. esculenta*) have decreased in abundance in response to recent warming (Simkanin et al. 2005; Brodie et al. 2009; Birchenough and Bremmer 2010). However, the evidence base is largely based on anecdotal reports and unpublished survey data, and detailed historical examinations of distribution patterns are lacking.

As changes in the identity and abundance of habitat-forming species can have wide-ranging consequences for community structure and ecosystem functioning (Jones et al. 1994), there is a pressing need to examine climate-driven distribution shifts and their wider implications. For example, if a cool-water habitat former is replaced by a warm water species that is functionally and structurally similar, it is plausible that the wider community or ecosystem will be relatively unimpacted (e.g., Terazono et al. 2012). Conversely, if a structurally or functionally dissimilar species becomes dominant or habitat formers are lost and not replaced, then widespread changes in biodiversity patterns and ecological processes are likely to ensue (Ling 2008; Thomsen et al. 2010). In the UK and Ireland, a range contraction of *A. esculenta*, the dominant species on very exposed shores and an important midsuccessional species in more sheltered locations (Hawkins and Harkin 1985), would impact community structure and functioning as there is no warm water equivalent. *Alaria esculenta* is particularly susceptible to climate fluctuations, having disappeared from much of the English channel during a warm period in the 1950s and not recovering as conditions became cooler in the 1960s (Southward et al. 1995). Replacement of *L. hyperborea* with *L. ochroleuca*, which are more similar both structurally and functionally, may have less knock-on effects, although subtle differences in kelp species traits have been shown to influence local biodiversity patterns (Blight and Thompson 2008). Most dramatically, the predicted increase in the relative abundance of *S. polyschides* (Birchenough and Bremmer 2010) could have major implications for kelp forest structure and functioning as it is a fast-growing, annual species with distinct morphological and ecological traits (Table 1). Similarly, increased abundance of another annual, *U. pinnatifida,* relative to perennial species would also represent a major ecological shift from a stable habitat to one dominated by boom–bust cycles, with significant knock-on effects for biodiversity and productivity (see Pedersen et al. 2005 for relevant fucoid example). As kelps make a significant contribution to coastal primary production, facilitate export of carbon from high to low-productivity systems, and fuel entire food webs, changes in the quality or quantity of detrital material resulting from climate-driven changes in kelp species identity, abundance, or productivity could have far-reaching consequences (Krumhansl and Scheibling 2012). In the UK and Ireland, the wider implications of shifts in kelp species identity and abundance for kelp forest productivity, trophic linkages, and ecosystem functioning are almost entirely unknown.

It may be possible to predict the future structure of kelp forests under continued ocean warming in the UK and Ireland by examining the current structure of kelp forests under warmer conditions further south. For example, coastal waters off northern Portugal are some ∼3°C warmer than off southern England and some ∼5°C warmer than in northwest Scotland, which is within the projected range of NE Atlantic warming within the next 50–80 years (Philippart et al. 2011). The structure of kelp forest habitats off northern Portugal and Spain is strikingly different from those in UK waters (Hawkins and Harkin 1985; Fernandez 2011; Tuya et al. 2012). Most obviously, the geographic range of *L. digitata* does not extend further south than France and therefore does not form dense stands in the low intertidal and shallow subtidal zones. *Laminaria hyperborea* is present southward to north Portugal, but is generally much smaller and lower in abundance, forming isolated patches rather than dense canopies under warmer conditions. Conversely, *L. ochroleuca* is more abundant and often larger, while *S. polyschides* is generally more abundant across a wider depth range. However, recent observations suggest that *S. polyschides* (Fernandez 2011; Diez et al. 2012; Voerman et al. 2013), *L. ochroleuca* (Fernandez 2011; Diez et al. 2012; Voerman et al. 2013), and *L. hyperborea* (Tuya et al. 2012; Voerman et al. 2013) have undergone range contractions and/or declines in abundance in recent decades in response to seawater warming along the Iberian Peninsula. Loss of canopy-forming macroalgae at large spatial scales will have major implications for biodiversity and ecosystem goods and services (Voerman et al. 2013). It is very likely that kelp forest biomass and productivity will be diminished under warmer, stormier conditions (Krumhansl and Scheibling 2012), although direct measurements of kelp forest structure, biodiversity, productivity, detritus production and export, and resistance and resilience to perturbation along a regional-scale temperature gradient along the NE Atlantic coastline are lacking. Comparative experimental work along regional-scale temperature gradients is a promising approach in climate change ecology and can yield critical information on the mediation of ecological processes by ocean climate (Wernberg et al. 2010, 2012). Comparative kelp research along a regional-scale temperature gradient along Western Europe, spanning from Portugal (average sea temperature ∼16°C) to Norway (average sea temperature ∼8°C), would significantly enhance our understanding of climate change impacts on kelp forest structure and functioning.

In conjunction with ocean warming, observed and predicted increases in storminess (Lozano et al. 2004; Weisse et al. 2005) and ocean acidification (Connell and Russell 2010; Koch et al. 2013) will also impact kelp forests. As canopy-forming macroalgae may be damaged and dislodged during periods of intense wave action (De Bettignies et al. 2013), increased storminess will affect the structure and functioning of entire kelp habitats, by altering patch dynamics (Dayton and Tegner 1984) and potentially driving ecological phase shifts (Dayton et al. 1999; Wernberg et al. 2011). With regard to ocean acidification, experimental work on noncalcifying macroalgae lags considerably behind research focussed on calcifying algae and invertebrates (Connell and Russell 2010; Wernberg et al. 2012), but some generalized responses are emerging. From a physiological viewpoint, noncalcifying fleshy algae such as kelps can utilize elevated CO_2_ concentrations to increase growth rates (Harvey et al. 2013; Koch et al. 2013; Kroeker et al. 2013) and, probably, increase thermal optima for key physiological processes to potentially offset the impacts of increased temperature (Koch et al. 2013). Thus, increased CO_2_ concentrations may benefit kelp species. However, from an ecological viewpoint, the competitive balance between kelps and noncalcifying turf-forming algae may be shifted toward the latter in a high CO_2_ world (Connell and Russell 2010). When kelp canopies are removed under conditions of thermal stress, poor water quality, or intense wave action, mats of turf-forming ephemeral algae can replace them to form an alternative, degraded habitat type. Under certain conditions, including poor water quality (see “Land–sea interface” section), turfs can persist in space and time to inhibit kelp recruitment and consequently restrict kelp forest recovery. Experimental evidence and predictive theory both suggest that turf-forming algae will prosper under elevated temperature and CO_2_ (Connell and Russell 2010), increasing the likelihood of large-scale shifts from structurally diverse kelp canopies with associated calcified and noncalcified flora to simple habitats dominated by noncalcified, turf-forming seaweeds. The ramifications of such shifts are far-reaching and include regional biodiversity patterns, trophic linkages, nutrient cycling, and habitat provision for socioeconomically important marine organisms (e.g., fish and crustaceans).

Finally, two key knowledge gaps concerning the climate change ecology of kelp forests. First, there is a paucity of information on the capacity of local kelp populations to acclimatize or even adapt to climate-mediated change. It is clear that kelp populations can maintain physiological processes under a wide range of environmental conditions through local adaptation (e.g., Delebecq et al. 2013), but the rate at which kelp species can respond to rapidly changing temperatures and other localized stressors is unclear. Second, seaweed populations are particularly susceptible to short-term extreme warming events (Dayton and Tegner 1984; Smale and Wernberg 2013; Wernberg et al. 2013), which may increase in magnitude and frequency as a consequence of anthropogenic climate change (Jentsch et al. 2007; Feng et al. 2013). Short-term climate variability may pose greater threat to kelp populations at lower latitudes (i.e., toward range edges) than those within midlatitude temperate regions. For example, southerly distributed kelp forests off Spain and Portugal, which are subjected to environmental variability driven by the strength of coastal upwelling, comprise species at thermal maxima with dynamic range edges (Fernandez 2011; Tuya et al. 2012; Voerman et al. 2013). Anomalous warming events also have the potential to cause stepwise changes in the structure and functioning of kelp forests in midlatitude systems, and greater understanding of the resistance and resilience of kelp populations and their associated communities to such events is of ever-growing importance. Moreover, the effects of short-term temperature variability will likely be compounded by additional simultaneous stressors, such as nutrient loading, pollution, disease, or fishing pressure, which may interact with extreme climatic events to reach ecological tipping points (Crain et al. 2008).

### Land–sea interface

As macrophytes are restricted to the photic zone, kelp forests form nearshore, coastally fringing habitats that are strongly influenced by connectivity between land and sea. Light is well known as the main driver of the distribution, depth, and abundance of kelp (Kain 1979; Dayton 1985), and contemporary declines in water clarity associated with coastal urbanization and land use have impacted macroalgal-dominated habitats across Europe (see Airoldi and Beck 2007 for review). Human activities across much of the world's temperate coastlines have increased sediment and nutrient loading into the coastal environments, which has been consistently linked with the widespread disappearance of kelp forests (e.g., Eriksson et al. 2002; Connell et al. 2008). Burrows (2012) recently showed that the distribution of *L. hyperborea* in the UK is strongly linked with ocean color (indicative of both oceanic phytoplankton content and terrestrially derived material), as greater light attenuation results in decreased depth penetration and abundance of kelp species and their associated communities. Off the coast of Norway, a recent large-scale disappearance of *S. latissima*, which has been replaced by ephemeral turfing algae, has been attributed to chronic eutrophication combined with increased temperatures (Moy and Christie 2012), although further work is needed to clarify these mechanisms. Clearly, processes acting across the land–sea interface can detrimentally impact the structure and functioning of kelp forests, and sustainable management of these habitats depends on integrated approaches spanning multiple ecosystems. In the NE Atlantic, these impacts will likely be exacerbated by both climate change, as precipitation rates and extreme climatic events are projected to increase (Philippart et al. 2011), thereby enhancing runoff, and by continued coastal development and land use.

Crucially, multiple concurrent stressors (climate and non-climate-related) do not act in isolation, but often combine synergistically in their effects, so that the total impact is far greater than the sum of individual factor effects (Crain et al. 2008; Harvey et al. 2013). Synergism can cause “ecological surprises”, where unexpected regime shifts occur quickly because a tipping point is exceeded (Crain et al. 2008). In kelp forests, multiple stressors can cause shifts from complex, biologically diverse habitats to simple turf-dominated “barrens” (Dayton and Tegner 1984; Ling et al. 2009; Russell et al. 2009). It is evident that increased nutrient loading and turbidity can interact with climate change factors to increase the competitive ability of ephemeral turf species, which can form an alternative stable state and inhibit the recovery of kelp forests (Russell et al. 2009; Moy and Christie 2012). The effects of multiple stressors on temperate algal communities are, however, poorly understood as only 20% of marine climate change experiments have focussed on primary producers and most have been single-factor laboratory experiments comprising few species (Wernberg et al. 2012). Continued research effort addressing the interactive effects of multiple climate and non-climate-related stressors under both laboratory and field settings should remain a priority.

### Top-down” processes

Overgrazing by invertebrate herbivores, particularly sea urchins, can decimate kelp forests and cause phase shifts from structurally and biologically diverse habitats to depauperate “barrens” (reviewed by Steneck et al. 2002). In the North Atlantic, the green sea urchin *Strongylocentrotus droebachiensis* has deforested extensive areas of kelp forest in eastern Canada (Mann 1977), Iceland (Hjorleifsson et al. 1995) and northern Norway (Leinaas and Christie 1996), with major consequences for ecosystem structure and functioning (Steneck et al. 2002). At lower latitudes, the importance of grazing by the purple sea urchin *Paracentrotus lividus* on macroalgal assemblages has been recognized along Mediterranean and Atlantic coastlines (Bulleri et al. 1999; Hereu et al. 2004; Tuya et al. 2012).

In the UK and Ireland, the extent of deforestation by urchin grazing is generally restricted and patchy, although heavily grazed areas are more common in Scotland. Urchin grazing can certainly be important in setting local distributions of macroalgae, including kelps. Some of the earliest grazing work was done in the Isle of Man (Jones and Kain 1967), which showed that the edible sea urchin *Echinus esculentus* may determine the lower depth limit of *L. hyperborea* stands through intense grazing of young sporophytes. Similarly, *P. lividus*, which is relatively common along the west coast of Ireland, influences the distribution of macroalgae within Lough Hyne through grazing activity (Norton 1978; Kitching and Thain 1983). Recent resurveys of Lough Hyne have suggested that since classification as a marine reserve in 1981, the abundance of several urchin predators (i.e., crabs and sea stars) has increased, leading to declines in *P. lividus* abundance and consequent changes in macroalgal assemblages (O'Sullivan and Emmerson 2011). The green sea urchin *Strongylocentrotus droebachiensis*, which is only found in the north of Scotland, may also cause restricted patchy deforestation, but extensive barren formation has not been attributed to this species.

### Harvesting and cultivation

The demand for kelp for human consumption, alginate production, aquaculture feed, and (potentially) biofuel has increased in recent decades and will almost certainly continue to grow. Direct removal of kelps has major implications for kelp population structure, whole community dynamics, and wider ecosystem functioning (Christie et al. 1998; Vásquez 2008; Krumhansl and Scheibling 2012). There is some evidence to suggest that due to the rapid recruitment and growth of kelps and their associated species, industrial-scale wild harvesting of kelps can be achieved sustainably. For example, in both Norway and Chile, some 130,000–200,000 tonnes is extracted annually and has been for some time (Vásquez 2008; Vea and Ask 2011). However, while a limited natural harvest may be sustainable if properly managed with appropriate fallow periods, the potential for impact on the other services provided by kelp may be considerable. Although kelps recruiting into harvested areas may reach preperturbed densities and sizes within a few years, their associated assemblages may take considerably longer to recover (Christie et al. 1998). Kelp harvesting also negatively impacts the abundance of gadoid fishes and reduces the area of habitat preferred by foraging seabirds (Lorentsen et al. 2010), for example.

Across Europe, the potential for kelp biomass to be used for conversion to biofuels has reignited interest in large-scale kelp harvesting. A realistic contribution to energy markets through bioethanol production may require more kelp than can be wild harvested from natural habitats, prompting efforts to develop methods of farming kelp. Mariculture of kelps is commonplace in Asia, particularly in China, where demand for seaweeds for human consumption is high. It is clear that intense kelp farming can impact local patterns of water movement and may cause organic enrichment of sediments and anoxia (Krumhansl and Scheibling 2012). However, many researchers are championing integrated aquaculture practices that utilize seaweeds as biofilters within multitrophic farming operations (Neori et al. 2004; Troell et al. 2009). In northwest Scotland, for example, cultivation of kelps and other seaweeds adjacent to salmon farms can generate significant yields of algal biomass while simultaneously removing waste nitrogen (Sanderson et al. 2012). However, the impacts of large-scale kelp cultivation in nonenriched systems are poorly known and may be detrimental. The Crown Estate recently commissioned an independent investigation into the wider ecological effects of proposed seaweed mariculture off the west coast of Scotland (Aldridge et al. 2012). Using ecosystem-based modeling approaches, the authors concluded that; *the effects of the proposed farming activity on nutrient concentrations are expected to be ‘marginally significant’……and might become ‘certainly significant’……The observable effects of nutrient removal would be a lower nutrient concentration in the water, decreased productivity and energy fluxes through the pelagic system, decreased flux of organic material to the seabed, and subtle alteration to community structure*. (Aldridge et al. 2012). It is beyond doubt that large-scale kelp production, through both wild harvesting and mariculture, has the potential to impact kelp populations, their associated benthic communities, and wider ecosystem structure and functioning. While it is recognized that a conservative ecosystem-based management approach is a prerequisite for achieving sustainable production, the baseline knowledge on the structure and functioning of kelp ecosystems at regional scales needed to underpin such an approach is currently lacking.

## Conclusions

Global emissions of greenhouse gases are tracking the high emission scenarios considered by the IPCC, suggesting that future climate impacts will be more severe than widely acknowledged in policy (New et al. 2011). A robust appreciation of the likely ecological consequences of climate change is therefore increasingly urgent. Moreover, coastal ecosystems, dominated by highly productive sea grass and macroalgal habitats, provide ecosystem services valued at ∼US$19,000 ha^−1^·year^−1^, making them the third most productive systems globally in terms of value per hectare (Costanza et al. 1997). In the UK alone, the estimated direct economic value of coastal marine ecosystems exceeds £15 billion per year (Beaumont et al. 2008). As such, any changes in structure and functioning, either as a result of the direct effects of anthropogenic change on ecologically important species or through climate-mediated changes in the strength and direction of ecological processes, could lead to broad-scale implications for the goods and services coastal ecosystems provide. There is a paucity of regional-scale species distribution data from the UK and wider NE Atlantic, especially for subtidal rocky reef habitats, which hinders our ability to detect ecological change at relevant spatial scales. Such information, when combined with experimental studies of the effects of climate warming and predictive modeling approaches, will allow us to describe and forecast responses to environmental change and human activities such as harvesting with greater confidence.

Pre-1980s, the marine biological community of Britain and Ireland significantly contributed to the wider understanding of kelp forest structure and function through world's leading research. However, in recent decades, following rising costs associated with scuba diving and shifts in research priorities, subtidal kelp-dominated habitats have been strikingly understudied despite their fundamental role in coastal food webs and ecosystems. In contrast, research on *Macrocystis* forests in California has yielded critical information on the relative importance of “top-down” versus “bottom-up” factors in structuring marine benthic communities (Foster et al. 2006; Halpern et al. 2006; Byrnes et al. 2011; Guenther et al. 2012), shed light on regional-scale variability in environmental drivers (Edwards 2004; Reed et al. 2011), and informed management actions such as the implementation of Marine Protected Areas (see White et al. 2011 and references therein). Similarly, intense field-based research on *Ecklonia* forests in Australia has yielded novel insights into scale dependency in species interactions (Irving and Connell 2006) and biodiversity patterns (Smale et al. 2010), the connectivity of populations (Coleman et al. 2011) and habitats (Wernberg et al. 2006), as well as the resilience of kelp forests to perturbations including increased herbivory (Ling 2008; Ling et al. 2009), short-term climate variability (Wernberg et al. 2013), and physical disturbance (Wernberg et al. 2010).

In the NE Atlantic, there is considerable scope for cutting-edge research on ecological resilience, functional ecology, and range-edge dynamics because (1) a number of habitat-forming kelp species co-exist, (2) some kelp species are found at the edge of their range, and (3) the region has warmed at rates above the global average. However, the current state of knowledge is poor, and even basic information on species distributions, kelp forest biodiversity, and species interactions is largely lacking. The current evidence base is largely anecdotal and entirely inappropriate for informing management decisions, while process-based knowledge acquired from realistic field-based observations and experiments is completely absent. We strongly urge that (1) funding agencies and marine management organizations acknowledge these knowledge gaps and provide the resources needed to begin to fill them, (2) researchers and institutions adopt the collaborative approach needed to share the financial and logistical burden of conducting subtidal field-based research, and (3) researchers develop close alliances with kelp ecologists in knowledge-rich regions (e.g., Australasia and North America) to adopt contemporary, cross-disciplinary approaches to kelp forest research in the NE Atlantic, which will expedite progress and facilitate comparative work across contrasting systems. In addition, shifts in occupational health and safety culture and an ever-growing institutional fear of litigation in the UK (and more recently in other research-intensive nations) have led to a disparity between the actual risk associated with scientific diving and the expenditure and resources deemed necessary to make scientific diving “safe”. Increased costs associated with training, personnel, and paperwork requirements – combined with greater allocation of funds to desktop data-mining exercises and hi-tech “omics” research relative to field-based marine ecology – have made scientific diving for ecological research almost unfeasible. Engaging in rational evidence-based discussion relating to actual (rather than perceived) risks associated with subtidal field work, and re-assessing health and safety and legal requirements accordingly, would allow more marine ecologists to get “wet” and facilitate real-world observations of coastal marine ecosystems. Only by valuing and supporting field-based ecology can we make significant progress in understanding the resilience of kelp forests to rapid environmental change, which is urgently needed to improve our ability to manage and conserve these important habitats.
